# What the Papers Say

**DOI:** 10.1093/jhps/hnv009

**Published:** 2015-02-14

*Journal of Hip Preservation Surgery (JHPS)* is not the only place where work in the field of hip preservation may be published. Although our aim is to offer the best of the best, we continue to be fascinated by work that finds it way into journals other than our own. There is much to learn from it so *JHPS* has selected six recent and topical articles for those who seek a brief summary of what is taking place in our ever-fascinating world of hip preservation. What you see here are the mildly edited abstracts of the original articles, to give them what *JHPS* hopes is a more readable feel. Thanks to Ajay Malviya (UK), *JHPS* Editorial Correspondent, for his hard work in bringing this section together. If you are pushed for time, what follows should take you no more than 10 minutes to read. So here goes …

## INFLUENCE OF CAPSULAR REPAIR VERSUS UNREPAIRED CAPSULOTOMY ON 2-YEAR CLINICAL OUTCOMES AFTER ARTHROSCOPIC HIP PRESERVATION SURGERY

Researchers in Chicago set up a prospective study to determine the influence of primary capsular repair on clinical outcome after arthroscopic hip preservation surgery at a minimum of 2-year follow-up [1].

Four hundred and three patients met the inclusion criteria and had complete data available, 235 in the unrepaired group (Group A) and 168 in the capsular repair group (Group B). All patients completed four patient-reported outcome (PRO) questionnaires preoperatively and at a minimum of 2-year follow-up. These included the Hip outcome score-activities of daily living (HOS-ADL) and HOS-sport-specific subscale (HOS-SSS) subsets, non-arthritic hip score (NAHS) and modified Harris hip score (mHHS).

The groups were unmatched in terms of age, body mass index (BMI) and gender with Group A having significantly older patients with higher BMI and Group B with a female preponderance. Group A patients also had greater chondral damage and lower preoperative PROs. The PROs significantly improved in both groups with Group B showing a significantly greater improvement in HOS-ADL and NAHS; however, after adjusting for confounding variables the significance was lost.

The authors concluded that capsular repair did not confer clinical superiority over the unrepaired capsulotomy group, although it did not negatively influence the results either.

## COMPLICATIONS ASSOCIATED WITH THE PERIACETABULAR OSTEOTOMY: A PROSPECTIVE MULTICENTER STUDY

The purpose of this North-American prospective multicenter study was to determine and categorize all complications associated with the periacetabular osteotomy performed by experienced surgeons [2].

The authors prospectively analysed perioperative complications in 205 consecutive unilateral periacetabular osteotomies performed at seven institutions by 10 surgeons. All perioperative complications were recorded at an average of 10 weeks and 1 year after surgery in standardized fashion using a validated complication grading system applied to hip preservation procedures. The mean patient age was 25.4 years. There were 143 female and 62 male patients.

Major complications (Grade III or IV) occurred in 12 patients (5.9%). Seven complications were evident at the 10-week visit and five at the 1-year visit. Nine of the complications required a second surgical intervention, including repair for acetabular migration or implant adjustment (four patients), incision and drainage for a deep infection (two patients), and heterotopic bone resection, contralateral peroneal nerve decompression, and posterior column fixation (one patient each). Three thromboembolic complications were managed medically. There were no vascular injuries, permanent nerve palsies, intra-articular osteotomies and/or fractures or acetabular osteonecrosis. The most common Grade-I or II complication was asymptomatic heterotopic ossification. Concomitant procedures most commonly included femoral osteochondroplasty (58%) or hip arthroscopy (20%), which could include labral repair or resection.

For surgeons experienced with the periacetabular osteotomy, it is a safe procedure but is associated with a 5.9% risk of Grade-III or IV complications beyond the learning curve. The majority of these complications resolved without permanent disability and one out of five patients require hip arthroscopic intervention to deal with labral tear.

## ANTEVERTING PERIACETABULAR OSTEOTOMY FOR SYMPTOMATIC ACETABULAR RETROVERSION: RESULTS AT 10 YEARS

Prof. Siebenrock’s group from Bern have reported their 10-year results in a previously described patient cohort that had corrective periacetabular osteotomy for the treatment of symptomatic acetabular retroversion [3].

Clinical and radiographic parameters were assessed preoperatively and at 2 and 10 years post-operatively. A Kaplan-Meier survivorship analysis of the 22 patients (29 hips) with a mean follow-up of 11 years (range, 9–12 years) was performed. In addition, a univariate Cox regression analysis was done with conversion to total hip arthroplasty as the primary end point and progression of the osteoarthritis, a fair or poor result according to the Merle d’Aubigné score or the need for revision surgery as the secondary end points.

The mean Merle d’Aubigné score improved significantly from 14 points (range, 12–17 points) preoperatively to 16.9 points (range, 15–18 points) at 10 years. There were also significant improvements with regard to hip flexion, internal rotation and adduction compared with the preoperative status. No significant increase of the mean Tonnis osteoarthritis score was seen at 10 years. The cumulative 10-year survivorship, with conversion to a total hip arthroplasty as the primary end point, was 100%. The cumulative 10-year survivorship in achievement of one of the secondary end points was 71% (95% confidence interval, 54–88%). Predictors for poor outcome were the lack of femoral offset creation and overcorrection of the acetabular version resulting in excessive anteversion.

The authors concluded that anteverting periacetabular osteotomy for acetabular retroversion leads to favourable long-term results with preservation of the native hip at a mean of 10 years. Overcorrection resulting in excessive anteversion of the hip and omitting concomitant offset creation of the femoral head-neck junction are associated with an unfavourable outcome.

## DIAGNOSIS AND 2-YEAR OUTCOMES OF ENDOSCOPIC TREATMENT FOR ISCHIOFEMORAL IMPINGEMENT

Surgeons from the hip preservation centre at Dallas, USA investigated the clinical and radiographic presentation of patients with ischiofemoral impingement (IFI) and assessed the outcomes of endoscopic treatment with partial resection of the lesser trochanter. The diagnosis of IFI was on the basis of physical examination tests provoking the impingement between the lesser trochanter and ischium and reproducible pain lateral to the ischium with the long-stride walking test. The presence of quadratus femoris muscle edema and a decreased ischiofemoral space on magnetic resonance imaging was also used to establish the diagnosis [4].

Five patients with IFI who underwent endoscopic treatment with partial resection of the lesser trochanter were retrospectively reviewed. The outcomes were assessed at a mean follow-up of 2.3 years (range, 2–2.5 years) through the mHHS and a visual analogue scale score for pain.

The mean mHHS increased significantly from 51.3 points preoperatively to 94.2 points at the final follow-up. The mean visual analogue scale score for pain significantly decreased from 6.6 before surgery to 1 at the final follow-up. The mean duration to return to sport after surgery was 4.4 months. No complication was observed.

The authors concluded that endoscopic treatment of IFI was effective at 2 years in patients with consistent clinical and imaging diagnostic findings.

## RESIDUAL DEFORMITY IS THE MOST COMMON REASON FOR REVISION HIP ARTHROSCOPY: A THREE-DIMENSIONAL CT STUDY

Research done at the University of Michigan, USA has attempted to confirm whether residual deformity is the commonest reason for revision hip arthroscopy. The purpose of this study was to define the three-dimensional (3D) morphology of hips with residual symptoms before revision femoroacetabular impingement (FAI) surgery and determine the limitation in range of motion (ROM) in these patients using dynamic, computer-assisted, 3D analysis [5].

The study includes 50 revision arthroscopic FAI procedures on 47 patients with residual FAI deformity and symptoms after prior unsuccessful arthroscopic surgery and compared with a control of 65 patients who underwent primary arthroscopic FAI procedure by the same surgeon during the study period. Three-dimensional models of the hips were created to allow measurements of femoral and acetabular morphology and ROM to bony impingement using a validated, computer-based dynamic imaging software. A comparison of the virtual correction with the actual correction in the primary successful FAI treatment cohort was performed. Correspondingly, a comparison of the recommended virtual correction with the correction evident at the time of presentation after failed primary surgery in the revision cohort was performed.

Ninety percent of patients undergoing revision surgery for symptomatic FAI had residual deformities; the mean maximum alpha angle in revision hips was 68° and was most often located at 1:15, considering the acetabulum as a clock face. Twenty-six percent of hips had signs of overcoverage with a lateral center-edge angle greater than or equal to 40°. Dynamic analysis revealed mean ROM before osseous contact was less in the revision group than those in the control group.

The authors concluded that marked radiographic evidence of incomplete correction of deformity was found on 3D reconstruction of CT scan images in patients with residual symptoms after FAI surgery and recommend careful attention to full 3D resection of impinging structures.

## DOES RADIOGRAPHIC COXA PROFUNDA INDICATE INCREASED ACETABULAR COVERAGE OR DEPTH IN HIP DYSPLASIA?

Researchers in Japan set out to determine the prevalence of radiographic coxa profunda in patients with hip dysplasia, the morphologic differences of the acetabulum and pelvis between patients with hip dysplasia and control subjects and the morphologic differences between hip dysplasia with and without coxa profunda [6].

The authors retrospectively reviewed the pelvic radiographs and CT scans of 70 patients with hip dysplasia. Forty normal hips were used as controls.

Normal hips were defined as those with a lateral center-edge angle between 25° and 40°. Coxa profunda was defined as present when the acetabular fossa was observed to touch or was medial to the ilioischial line on an anteroposterior pelvic radiograph. CT measurements included acetabular version, acetabular coverage, acetabular depth and rotational alignment of the innominate bone.

The prevalence of coxa profunda was 44% in dysplastic hips and 73% in the control hips (odds ratio, 3.32). Dysplastic hips had a significantly more anteverted and globally shallow acetabulum with inwardly rotated innominate bone compared with the control hips. Dysplastic hips with coxa profunda had significantly more anteverted acetabulum and inwardly rotated innominate bone compared with those without coax profunda, whereas the acetabular coverage and depth did not differ between the two groups, with the numbers available.

The authors concluded that radiographic coxa profunda was not a sign of increased acetabular coverage and depth in patients with hip dysplasia, but rather indicates classic acetabular dysplasia, defined by an anteverted acetabulum with anterolateral acetabular deficiency and an inwardly rotated pelvis. Thus, the presence of coxa profunda does not indicate a disease in addition to hip dysplasia, and the conventional maneuvers during periacetabular osteotomy are adequate for these patients.
